# Challenges of Monitoring the Gluten-Free Diet Adherence in the Management and Follow-Up of Patients with Celiac Disease

**DOI:** 10.3390/nu13072274

**Published:** 2021-06-30

**Authors:** Herbert Wieser, Ángela Ruiz-Carnicer, Verónica Segura, Isabel Comino, Carolina Sousa

**Affiliations:** Department of Microbiology and Parasitology, Faculty of Pharmacy, University of Seville, 41012 Seville, Spain; acarnicer@us.es (Á.R.-C.); vsegura@us.es (V.S.); icomino@us.es (I.C.)

**Keywords:** celiac disease, patients with CD, dietary adherence, gluten-free diet, symptoms

## Abstract

Celiac disease (CD) is a chronic gluten-responsive immune mediated enteropathy and is treated with a gluten-free diet (GFD). However, a strict diet for life is not easy due to the ubiquitous nature of gluten. This review aims at examining available evidence on the degree of adherence to a GFD, the methods to assess it, and the barriers to its implementation. The methods for monitoring the adherence to a GFD are comprised of a dietary questionnaire, celiac serology, or clinical symptoms; however, none of these methods generate either a direct or an accurate measure of dietary adherence. A promising advancement is the development of tests that measure gluten immunogenic peptides in stools and urine. Causes of adherence/non-adherence to a GFD are numerous and multifactorial. Inadvertent dietary non-adherence is more frequent than intentional non-adherence. Cross-contamination of gluten-free products with gluten is a major cause of inadvertent non-adherence, while the limited availability, high costs, and poor quality of certified gluten-free products are responsible for intentionally breaking a GFD. Therefore, several studies in the last decade have indicated that many patients with CD who follow a GFD still have difficulty controlling their diet and, therefore, regularly consume enough gluten to trigger symptoms and damage the small intestine.

## 1. Introduction

Celiac disease (CD) is a chronic T-cell-mediated enteropathy caused by dietary exposure to the storage proteins of wheat, rye, barley, and some varieties of oats (called gluten in the field of CD) in genetically predisposed individuals [1,2,3,4]. Epidemiological data suggest a prevalence of approximately 1% in the general population of Western countries, Australia and New Zealand, but CD is also present in North Africa and major parts of Asia. To date, CD occurs rarely in people from other parts of Sub-Saharan Africa [5]. The precipitating gluten comprises hundreds of different proteins, which are roughly divided into the alcohol-soluble prolamins and the alcohol-insoluble glutelins [6]. Gluten proteins have been given the following cereal-specific names: wheat gliadins (prolamins) and glutenins (glutelins), rye secalins, barley hordeins, and oat avenins. They are all structurally characterized by unique repetitive amino acid sequences, rich in glutamine and proline, which are commonly considered the triggering factor of CD [7]. In particular, the high proline content makes these proteins resistant to complete digestion to ensure that long-chain immunogenic peptides reach the intestinal mucosa.

The pathogenesis of CD consists of the CD-specific passage of immunogenic gluten peptides through the small intestinal epithelium, and the combined adaptive and innate immune responses to the peptides in the lamina propria [2,8]. CD predominantly affects the duodenal intestine and induces a general flattening of the mucosa characterized by villous atrophy, crypt hyperplasia, and increased lymphocyte infiltration of the epithelium [2,9,10,11,12,13]. Moreover, CD is marked by a disease-specific antibody response to gluten peptides and tissue transglutaminase (autoantibodies). In addition to the ingestion of gluten and genetic predisposition, environmental factors such as infections, imbalanced intestinal microbiota, and increased intestinal permeability have been associated with the development of CD [4,14,15]. The clinical presentation of CD is extremely variable and can be divided into intestinal symptoms such as chronic diarrhea, abdominal pain, and, among children, the failure to grow normally as well as extra-intestinal manifestations including conditions caused by deficiencies of essential nutrients, neurological disorders, psychiatric complaints, dental enamel defects, liver abnormalities, joint manifestations, dermatitis herpetiformis, bone disease, problems in reproductive and endocrine systems, etc. [1,16,17]. A considerable number of patients present with atypical symptoms or even no symptoms despite the presence of a flattened small intestinal mucosa and CD-specific serum antibodies (asymptomatic CD) or present only CD-specific serum antibodies (potential CD). The diagnostic scheme of CD is usually based on clinical history and the presence of symptoms typical of CD, testing of CD-specific serum antibodies, histological judgement of small intestinal biopsies, and response to a gluten-free diet (GFD) [1,2]. In young children with clear symptoms and positive serology (10× the upper limit for normal antibody levels), CD diagnosis may be established without histological examination according to the diagnostic criteria of the European Society of Pediatric Gastroenterology, Hepatology, and Nutrition (ESPGHAN) [18,19].

Following a GFD creates difficulties and limitations in the life of patients with CD. Therefore, non-adherence to a GFD is a daily occurrence, which delays or prevents patient’s healing. Compliance with a GFD among patients with CD, examined in the last few decades, is in the range of 45 to 90% [20]. Inadvertent gluten intake occurs more frequently than intentional intake, and gluten contamination in naturally or certified gluten-free foods and meals is likely to be one of the most important factors of inadvertent non-adherence to a GFD [21,22]. However, a strict GFD usually results in prompt relief of clinical symptoms, while recovery of small bowel mucosal damage may even take years [23]. A strict GFD is currently indicated in all cases of symptomatic CD and has also been recommended for asymptomatic patients. Recently, Ruiz-Carnicer et al. [24] have demonstrated that the fact that patients remain asymptomatic does not imply that they have not consumed gluten and that they are not at risk of developing histological lesions and complications as a result of their condition. In contrast, whether patients with potential CD should be treated with a GFD remains unclear [25].

This review focuses insight into the problematic issues of adherence to a strict GFD in patients with CD. Apart from highlighting the celiac dietary adherence methods, the recent literature on monitoring and the rate of GFD adherence, as well as on barriers to adherence, are presented.

## 2. Adherence to a GFD

Permanent, lifelong adherence to a strict GFD is the only available treatment for CD. Traditional cereal-based gluten-containing foodstuffs such as bread, pasta, and beer must be replaced by corresponding surrogates made from raw materials that do not contain gluten. However, a lifelong strict GFD is not easy, due to gluten ubiquity, cross-contamination of foods, improper labeling, and social constraints [20,26] and, therefore, a considerable portion of patients with CD do not adhere to a GFD. Numerous studies have investigated the factors influencing the compliance to a GFD, showing that adherence rates in patients with CD are well below optimal. A systematic review, summarizing the literature between 1980 and 2007, on the adherence to a GFD, had the following important findings: Rates for strict adherence ranged from 42 to 91%, depending on definition and method of assessment [27]. Adherence was most strongly associated with cognitive, emotional, and socio-cultural influences, membership to an advocacy group, and regular dietetic follow-up. Recent developments, including methods for monitoring adherence, the recently determined degree of adherence, understanding reasons for non-adherence, and interventions to improve adherence are outlined in the subsequent sections.

### 2.1. Monitoring Adherence

After diagnosis, it is important to monitor the adherence to a GFD to prevent ongoing symptoms and small intestinal damage. Non-responsiveness to a strict GFD, i.e., the presence of ongoing symptoms, could be caused by refractory CD or other complaints such as irritable bowel syndrome, lactose intolerance, or gastroesophageal reflux disease apart from non-adherence to a GFD [28,29,30,31]. Therefore, monitoring adherence is essential for identifying the cause on the ongoing symptoms. The following several procedures involving various approaches have been employed [32]: (a) periodic visits by expert nutritionists, (b) structured questionnaires, (c) clinical follow-up, (d) CD-specific antibodies, (e) gluten detection technologies that measure gluten prior to consumption in food samples, (f) gluten immunogenic peptides (GIP) in stools and/or urine, (g) serial endoscopies with collection of duodenal biopsies, and (h) and other endogenous markers such as fecal calprotectin (FC) measurements.

An endoscopy to collect intestinal biopsies is an invasive, expensive, and impractical procedure for frequent monitoring of GFD compliance. Additionally, since it may take up to two years for complete histological resolution of CD-related intestinal lesions, there is only a modest correlation between intestinal histology and the assessed dietary adherence that can be observed [33]. Similarly, symptomatic improvement during clinical follow-up may not accurately indicate adherence to a GFD because patients with proven strict adherence show more symptoms than healthy subjects [34]. Moreover, persistent symptoms may be induced, for example, by small-intestinal bacterial overgrowth, irritable bowel disease, microscopic colitis, and refractory CD, and therefore, are not markers for non-adherence. Additionally, a number of patients are asymptomatic, despite CD-specific small intestinal atrophy [23,35]. FC is used to diagnose and monitor inflammatory bowel diseases, such as ulcerative colitis and Crohn’s disease, as well as in the differentiation of functional and organic intestinal pathologies [36]. The evidence currently shows a correlation between FC and CD activity in the pediatric population; however, there is a lack of studies in adult patients with CD [37,38].

#### 2.1.1. Follow-Up by a Dietician

Studies have shown that patients who receive individual instructions on gluten-free foods and a GFD from healthcare providers are more likely to adhere to a GFD. Adherence is assessed by dietetic interviews supplemented by dietary questionnaires, such as the Standardized Dietician Evaluation (SDE). Measuring GFD adherence through patients´ self-reporting appears to be subjective and less accurate because it relies on the patient´s possibly limited knowledge of a GFD and gluten-free foods. In 2009, a simple validated Celiac Dietary Adherence Test (CDAT) for adults with CD was developed, which is one of the few validated measures available [39]. Items and domains, believed to be essential for successful GFD adherence, were used to develop an 85-item survey, which was administered to 200 individuals with biopsy-proven CD, who underwent standardized dietician evaluation and serological testing. A compacted 7-item questionnaire proved to be clinically relevant, easily administered, correlated highly with the SDE, and performed significantly better than serological testing [40].

A fast questionnaire, based on four simple questions with a five-level score (0–4; the Biagi score) was also shown to be a reliable and simple method of verifying adult patient compliance with a GFD [41]. The questionnaire was administered to 141 adult patients with CD on a GFD who were undergoing re-evaluation [42]. The score obtained was compared with the persistence of both villous atrophy and endomysial antibodies (EMAs). The rate of lower scores was higher among patients with the persistence of either villous atrophy or positive EMAs. For pediatric patients, a study of 151 children with CD demonstrated that short dietary questionnaires detected dietary transgressions only in 14% of patients, while a standardized dietary interview substantiated non-adherence in 52% of patients (Table 1) [43].

However, there is considerable controversy regarding the validity of dietary questionnaires in assessing a GFD because patients do not intentionally record actual gluten consumption in the questionnaire. At the same time, there is evidence suggesting that the intervention of expert nutritionists cannot aid the detection of exposures in ~30% of the patients who present with mucosal damage until up to a third biopsy [71].

#### 2.1.2. Serological Testing

The analysis of CD-specific serum antibody levels is a useful diagnostic tool in clinical practice and plays a supporting role in monitoring dietary compliance [72]. Indeed, there is evidence that persistently elevated levels of serum antibodies against gliadin (AGAs), transglutaminase 2 (TGAs), or deamidated gliadin peptide (DGPAs) can indicate non-adherence to a GFD. The performance of four different antibody collections (IgA DGPA, Ig A+G DGPA, IgA TGA, and IgA AGA) in detecting compliance with a GFD was tested in 95 Italian CD children with CD on a GFD >1 year [73]. Adherence interviews and serum collections were performed every three months. The Ig A+G DGPA level seemed to be the best for monitoring compliance with a GFD. The sensitivity to (i.e., ability to detect) transgressions from a GFD was 100% at 9 to 12 months and decreased to 76% after more than 1 year on a GFD. The IgA TGA and IgA AGA sensitivities were much lower (24 and 4%, respectively). To evaluate compliance with a GFD in a clinical ambulatory setting, a rapid IgA TGA assay, based on a whole-blood drop, was tested and compared with a conventional Enzyme-Linked ImmunoSorbent Assay (ELISA) and the patients´ interviews [74]. The results showed that the rapid test was just as reliable as a conventional ELISA and easy to perform in the ambulatory setting. However, patient interviews were shown to be more sensitive than serology in identifying patients who do not adhere to a GFD.

However, the normalization of antibody titers takes a long time, and these tests cannot identify incidents of occasional gluten exposure. Therefore, their use is limited to indicating a lack of adherence but is of no value for evaluating whether there is strict adherence to a GFD. Moreover, up to 10% of patients with CD are seronegative, despite the positive histology of duodenal biopsy samples. In 2007, a prospective comparative study, including 154 adult patients with CD on a GFD, demonstrated that serological tests cannot replace evaluations by trained nutritionist evaluation in the assessment of GFD adherence [75]. More recently, a comparison with a standardized evaluation by a registered dietician revealed that negative TGA levels are not necessarily indicative of good adherence to a GFD in pediatric patients with CD [76].

Altogether, the data clearly show that serology and dietary questionnaires at follow-up have a poor correlation with mucosal healing and, therefore, relying solely on these may underestimate the activity of CD [24,45,46,60,68,69,77,78,79].

#### 2.1.3. Stools and Urine Testing

GIP, analyzed in stools and urine by using commercial ELISAs (monoclonal antibodies G12 and/or A1), have been proposed as new non-invasive biomarkers to detect gluten intake and to verify GFD compliance in patients with CD [45,46,49,80,81,82,83,84,85]. GIP are resistant to gastrointestinal digestion and responsible for immunogenic reactions in the T cells of patients with CD [83]. Unlike traditional methods for monitoring GFD adherence, which only evaluate the consequences of GFD transgressions, this non-invasive method enables a direct and quantitative assessment of gluten exposure.

These simple immunoassays could overcome some key unresolved scientific and clinical problems in CD management. Several prospective studies have been carried out to investigate the efficacy of GIP determination in stools. To assess the capacity to determine gluten ingestion and to monitor GFD compliance in patients with CD by the detection of GIP equivalents in stools, 53 children with CD (age range: 1–12 years) were enrolled [82]. Seven subjects had active disease and 46 subjects maintained a GFD for >2 years. After the controlled ingestion of a fixed amount of gluten (9–30 g), stools samples were analyzed using a G12 competitive ELISA. The results demonstrated that the method was a reliable tool for the detection of GFD transgressions in patients with active CD and CD in remission. A prospective multicenter study, including 188 patients with CD on a GFD and 73 healthy controls on a gluten-containing diet, revealed that 56 patients with CD (30%) had high levels of GIP in their stools [45]. All the controls except one (98.5%) had quantifiable amounts of GIP in stools. The results for patients with CD showed increasing dietary transgressions with advancing age (39% over 13 years) and gender (a predominance of males in this evaluation). Simultaneously, the study indicated limitations of traditional methods, such as food questionnaires or serological tests, for monitoring a GFD in patients with CD (Table 1).

To investigate the course of gluten intake after a diagnosis of CD and subsequent GFD, the stools of 64 pediatric patients with CD were analyzed for GIP at diagnosis and 6, 12, and 24 months thereafter [49]. Most of the children (97%) had detectable GIP at diagnosis. After GFD initiation, the rate of children with detectable GIP decreased to 13% at 6 months and increased to 25% at 24 months (Table 1). A recent examination of 25 patients with CD on a GFD for at least one year revealed that four patients (16%) tested positive for stool GIP [86]. Two of them complied strictly with a GFD according to the Biagi questionnaire and none of them manifested symptoms. The results demonstrated that stool GIP analysis identified those patients who did not comply with a GFD more accurately than a validated questionnaire. Therefore, monitoring the GIP in stools offers a direct objective quantitative assessment of exposure to gluten after CD diagnosis.

To compare the sensitivity and specificity of a rapid lateral flow technique (LFT) based on G12 and A1 monoclonal antibodies with the G12 ELISA method, stool samples from 54 healthy infants divided into two groups were analyzed [85]. Group 1 included infants aged 6 to 24 months, with an unrestricted consumption of gluten-containing cereals. Group 2 (negative controls) comprised of infants aged 0 to 6 months, either breastfed or formula fed, who had never ingested gluten. In group 1, all the infants had positive values using a conventional ELISA, while the LFT was negative in 5/20 cases. In group 2, all the samples were negative using both methods. Therefore, both methods were highly specific, while an ELISA had a higher sensitivity.

Urine can also be used to monitor GFD adherence, as shown in the following studies. A total of 76 healthy individuals (aged 3–57 years) (group 1) and 58 patients with CD (aged 3–64 years) (group 2) were subjected to different dietary conditions [46]. Urine samples were collected, concentrated, and analyzed for the presence and quantities of GIP by means of an LFT based on G12 antibodies. GIP were detectable in concentrated urines from group 1 individuals (previously subjected to a GFD) as early as 4–6 h after gluten intake (ingestion of at least a portion of pasta, bread, or whole grain from wheat, barley, and rye) and remained detectable for 1–2 days. The experiments indicated that the ingestion of >25 mg gluten could be detected in urine. The presence of GIP in the urine of group 2 (patients with CD on a GFD) revealed a high percentage of non-compliance with a GFD. GIP in urine were detectable in 48% of adults and 45% of children. An examination of duodenal biopsies revealed that most of the treated patients with CD without villous atrophy (89%) had no detectable GIP in their urine (Table 1), while all the patients with quantifiable GIP in their urine showed an incomplete intestinal mucosa recovery [46].

A simple and highly sensitive point-of-care (POC) device, based on a surface plasmon resonance biosensor and G12 and A1 monoclonal antibodies, enabled the rapid and efficient monitoring of a GFD directly in urine [87,88]. The excellent limits of detection of GIP (1.6–4.0 ng/mL) ensured the detection of gluten intake around the maximum amount tolerable for patients with CD (<50 mg). No sample pre-treatment, extraction, or dilution was required, and the analysis took less than 15 min. The assays had an excellent reproducibility (coefficient of variation: 3.6 and 11.3% for G12- and A1-based assays, respectively) and were validated with real samples.

Commercial ELISA (stool) and LFT (stool and urine) kits were used in parallel to assess the excretion of GIP in stools and urine [64]. Forty-four patients with CD, following a GFD >2 years, were asked to deliver stools and urine samples and were examined twice, 10 days apart. Considering the results for both assays, 11 patients (25%) had at least one positive GIP test. The ELISA and LFT were concordant (concomitantly positive or negative) in 67 out of 74 stools samples. To examine how often subjects with CD are still exposed to gluten, 53 Argentinian patients, who had been on a GFD for >2 years, collected stool each Friday and Saturday, and urine each Sunday for 4 weeks [70]. GIP were measured using a conventional ELISA (stool) and an LFT (urine). Overall, 159 of 420 samples (38%) were positive for GIP; 89% of patients had at least one positive stool or urine sample. On weekends, 70% of patients excreted GIP at least once compared with 62% during weekdays (Table 1).

Recently, an article by Ruiz-Carnicer et al. [24] provided additional data supporting the utility of GIP testing in the management of CD. The authors found that 58% of the patients with CD consuming a GFD had detectable GIP in their urine at least once, with higher rates of positivity on the weekend. The results demonstrated the high sensitivity (94%) and negative predictive value (97%) of GIP measurements in relation to duodenal biopsy findings. Additionally, they demonstrated that 25% of the patients on a GFD presented with Marsh type II–III duodenal lesions. If the authors had only considered serology, symptoms, or questionnaire scores, and had not performed a duodenal biopsy, 60–80% of these patients would have been overlooked. It was demonstrated that taking a GIP measurement on three days of the week, including the weekend, could be the best option to confirm GFD adherence in the short term and appears to accurately predict the absence of histological lesions. The introduction of GIP testing as an assessment technique for GFD adherence may help in ascertaining dietary compliance and to target the most suitable intervention during follow-up. Additionally, this would eliminate the uncertainty in predicting the reappearance of villous atrophy and its possible complications and the association between any nonspecific symptomatology and the underlying disease.

Moreover, a key future use of GIP may be the evaluation of nonresponsive CD. Since refractory CD type 1 is a diagnosis of exclusion based on a traditional dietary adherence assessment, the use of GIP could reveal unsuspected gluten exposures in this population as well and guide an intervention. It may also help distinguish between gluten exposures and irritable bowel syndrome or fermentable oligosaccharides, disaccharides, monosaccharides, and polyols (FODMAPs) intolerance among symptomatic individuals. In addition, these new technologies help to improve not only healing, but also quality-of-life outcomes such anxiety and depression in patients with CD.

### 2.2. Rate of Adherence

Numerous international studies on GFD adherence rates in patients with CD have been published in the last few decades. Italian research groups have been leading in the investigation of GFD adherence. The rates of gluten-free adherence presented in the literature are characterized by extreme variability among the populations studied. For example, 38 studies, published up to 2007, indicated an adherence rate ranging between 42 and 91% [89]. More recently, compliance with a GFD has been reported to be in the range of approximately 45 to 90% [20]. In the case of children, a systematic review of 49 studies, published up to 2018, revealed adherence rates ranging from 23 to 98% (Table 1) [90]. The broad variability in adherence rates reported in the literature may be explained by the different populations examined (e.g., adults, adolescents, children, ethnic minorities), but also by the different methods used for determining adherence, the quality of investigations, and the definition of adherence (e.g., strict, high, partial, fair, poor adherent, or non-adherent). Despite the importance of monitoring the adherence to a GFD, there are no clear guidelines for how to explore this. In the following, selected studies from the last decade present data on adherence rates grouped into examinations of adults, adolescents, children, and ethnic minorities. The adherence rate was predominantly evaluated using a CDAT (Table 1).

Adults. A total of 65 Italian patients newly diagnosed with CD were re-evaluated after one year on a GFD [56]. According to dietary interviews, based on a CDAT, 82% had adequate adherence to a GFD. To evaluate differences in the GFD adherence between the clinically diagnosed and screening-detected Italian patients with CD, the medical records of 750 subjects, diagnosed during 2004 and 2013, were evaluated [63]. The patients were considered to have complied with a GFD, if all the following criteria were satisfied: (1) no reported intentional or accidental gluten ingestion; (2) absence of CD-related symptoms; and (3) negative IgA-TGA. The results clearly demonstrated that both groups of patients shared similar GFD adherence (91% versus 90%) years after the diagnosis. In another Italian investigation, 104 patients with CD took part in a study focused on the relationship between adherence to a GFD and knowledge of the disease and its treatment in general [65]. By means of a CDAT, 65% of patients reported strictly adhering to a GFD (Table 1). Adherence was strongly and significantly associated with knowledge of CD and a GFD.

A total of 271 Spanish patients with CD completed a series of questionnaires regarding adherence to a GFD among other items; 72% of subjects indicated an excellent or good adherence (Table 1) [67]. Higher levels of adherence were particularly associated with CD-specific self-efficacy. Three studies from the UK, which compared the GFD adherence of white and South Asian patients with CD, indicated that white patients were adherent to a GFD in a range from 53 to 81%. To examine the GFD adherence of patients with CD in Israel, 301 subjects completed an anonymous online questionnaire sent via the Israeli Celiac Association and social networks [66]. According to the Biagi score, 82% of patients were found to be highly adherent to a GFD (score 3–4) and 18% were poorly adherent (score 0–2) (Table 1). Young age at diagnosis and smoking were significantly associated with non-adherence to a GFD.

To assess GFD adherence among a Canadian community, 222 patients with CD on a GFD completed a CDAT [59]. The results revealed that the degree of strict adherence was only 56%. Another group of Canadian adults with CD was enrolled to examine GFD adherence six months after diagnosis by means of a CDAT [60]. Of the 105 participants, 91% reported gluten exposure less than once a month and thus were consistent with adequate adherence. To determine long-term GFD adherence, 355 US patients with CD were re-evaluated after a mean time of 9.9 years on a GFD [57]. Adequate adherence, determined using a CDAT, was found in 76% of the patients (Table 1).

The evaluation of GFD adherence among Mexican adults with CD (*n* = 56) and non-celiac gluten sensitivity (NCGS) (*n* = 24) using a CDAT revealed that 58% of subjects perceived themselves as strictly adherent [58]. However, inadvertent gluten intake was frequent in both CD (39%) and NCGS (33%). The result of a CDAT provided to 5310 adult and adolescent Australians and New Zealanders with CD, showed that 61% were adherent to a GFD (Table 1) [62]. Older age, being male, symptoms after gluten ingestion, better food knowledge, and lower risk of psychological distress were independent predictors of adherence. In summary, thirteen studies from nine countries indicated adherence rates among adult patients with CD in a range from 53 to 91%.

Adolescents. Concerning dietary compliance, 58 young Italian patients with CD around the transition age were asked to answer the question: “Do you voluntarily eat gluten-containing food?” Nobody answered, “often or at times”; 16 subjects answered, “on special occasions”; 21, “rarely”; and 21, “never” [54]. Out of the 21 patients who declared no dietary lapses, five showed positive serology, which indicated that they were underestimating or not aware of gluten contamination in food (Table 1).

To investigate the GFD adherence of 70 Swedish adolescents with CD detected by screening, they filled in a CDAT and came to a five-year follow-up [55]. The evaluation showed that 86% of the adolescents were adherent to a GFD five years after screening (Table 1).

The rate of non-adherence to a GFD among 35 patients with CD under 20 years of age was assessed in a tertiary Brazilian referral center by means of a questionnaire and a serological test [53]. Despite dietary guidance, 20% of the patients reported non-adherence to the diet. Altogether, three studies on adolescents from three countries revealed adherence rates from 36 to 86% (Table 1).

Children. A Polish study compared frequency and cause of diet failure in 102 children with CD treated with a GFD for >2 years [47]. Dietary adherence was evaluated serologically (TGA test) and using a questionnaire. The results showed that one-third of the patients, mainly children aged 13–18 years, failed to follow a GFD. Younger children (up to 12 years) were less likely to abandon the diet. In this age group, inadvertent diet failure prevailed, while teenagers predominantly interrupted their diet intentionally. Personal questionnaires, completed by 325 parents or caregivers of pediatric patients with CD from the Slovak Republic, revealed that strict GFD adherence was maintained by 69% of children [48]. Adherence was significantly higher among girls compared to boys, younger children, children with a family history of CD, and children of parents with higher education.

The GFD adherence of 200 Italian children with CD was assessed to evaluate differences as a consequence of transition from a referral center (V1) to a general pediatrician (V2) [52]. Adherence was measured using the Biagi score and the IgA TGA test at the last follow-up at V1 and at an annual follow-up at V2. Adherence at V2 was significantly worse compared with V1: 84% vs. 95% (Biagi score) and 97% vs. 100% (TGA test), respectively. A study of 134 Indian children with CD using a questionnaire-based interview showed that 88 patients (66%) were adherent to a strict GFD [44]. Compliance was higher in children up to 9 years of age than in children aged >9 years. In summary, four studies from four countries showed adherence rates of children in a range from 66 to 84% (Table 1).

Ethnic minorities. Differences in GFD adherence between ethnically different patients were reported in three studies from the UK. After CD diagnosis, 71 South Asian and 67 white adult patients with CD from a single center in Southern Derbyshire were advised to maintain a GFD [91]. After six weeks on a GFD, the patients were classified by an experienced dietician as adhering strictly to a GFD (not ingesting any gluten) or taking gluten. Fifty-four white patients (81%) had strict diet compared with only 37 Asians patients (52%). A combined cross-sectional survey, based on a CDAT and CD adherence score, was used to determine the GFD adherence of 375 white and 38 South Asian patients with CD residing in the UK [92]. The results demonstrated an almost identical rate of adhering to a GFD (53 and 52%, respectively). The examination of ethnically diverse populations with CD in the North West of England was performed using the assessment of dietetic notes from follow-up visits with dieticians [93]. The results revealed that the rate of strict adherence to a GFD in the 33 South Asian patients was significantly lower (12%) than that of the 113 tested Caucasian patients (65%). Altogether, the adherence rates of ethnic minorities in the UK, assessed in three studies, ranged from 12 to 52% (Table 1).

Few studies have tried to separate intentional and inadvertent gluten ingestion. Identification of inadvertent gluten consumption, for example, using questionnaires or interviews, is more difficult or even impossible compared to intentional consumption. Therefore, the rate of inadvertent non-adherence, caused by, for example, contaminated naturally gluten-free products or hidden vital gluten, is likely to be highly underestimated and this fact should be particularly considered in the judgement of reported GFD adherence rates. A cross-sectional survey on intentional and inadvertent non-adherence was conducted using a self-completion questionnaire received from 287 adult patients with CD from the North East of England [90]. Intentional gluten consumption was common (40%), but not as frequent as inadvertent lapses. A multicenter study, including seven Spanish hospitals, investigated the adherence of 366 adult patients with CD using the Morisky questionnaire scale [94]. Results showed that 71.5% of patients reported perfect treatment adherence, 23.5% inadvertent poor adherence, and 5% intentional poor adherence. A total of 82 Canadian patients with CD, having a medium of 6 years GFD experience, completed a questionnaire with items related to GFD information including GFD adherence [95]. They reported strict adherence (55%), inadvertent gluten consumption (21%), and intentional gluten consumption (18%). In summary, three studies from three countries clearly demonstrated that intentional non-adherence to a GFD was less frequent than inadvertent non-adherence (Table 1).

In conclusion, studies from the last decade indicated that many patients with CD following a GFD still have difficulties in controlling their diet and hence regularly consume sufficient gluten to trigger symptoms and small intestinal damage. The rates of GFD adherence did not significantly change compared to previous decades. Ethnic minorities appear to be at the highest risk for non-adherence. Inadvertent lapses are distinctly more frequent than intentional lapses.

### 2.3. Factors Influencing Dietary Adherence

A comprehensive understanding of the facilitators and barriers associated with a strict GFD adherence is needed to develop strategies and resources to assist patients with CD following a GFD. Causes of adherence/non-adherence to a GFD are numerous and multifactorial, but limited evidence is available on their nature and magnitude (recently reviewed by Muhammad et al. [20] and Abu-Janb and Jaana [26]). A number of factors governing long-term GFD adherence have been identified by Leffler et al. [33] and Villafuerte-Galvez et al. [57]. An overview of the recent literature on factors that may limit or enhance the GFD adherence of patients with CD is presented in the following paragraphs, including a consideration of gluten cross-contamination in foods, problems in gluten analysis, knowledge of a GFD and gluten-free foods as well as availability, costs, and quality of dietetic gluten-free foods and a broad spectrum of individual factors.

#### 2.3.1. Gluten Cross-Contamination

Gluten cross-contamination in gluten-free foods is omnipresent; therefore, it is complicated for patients with CD to maintain a zero-gluten diet. Cross-contamination contributes majorly to inadvertent non-adherence and affects both naturally and certified gluten-free foods and composite foods containing hidden gluten as an additive. Naturally gluten-free material can be contaminated with gluten-containing cereals in the field, during storage, transport, and food production. Usually, these foods are not analyzed for gluten and not labeled gluten-free, and thus, are virtually considered safe by patients with CD. However, a number of studies have demonstrated that naturally gluten-free foods can be heavily contaminated with gluten. For example, a pilot study revealed that some naturally gluten-free grains, seeds, and flours, used for gluten-free food production, contained gluten levels up to 2925 mg/kg [96]. Consequently, naturally gluten-free foods are major contributors to inadvertent GFD lapses, which can hardly be avoided.

Labeled dietetic gluten-free foods seem to be safe for patients with CD. Numerous investigations, however, have shown that gluten levels above the threshold of 20 mg/kg are a daily occurrence. For example, a systematic review including 24 cross-sectional studies revealed that, on average, 13% of industrial products labeled gluten- free and 42% of gluten-free products offered by food services exceeded the threshold of 20 mg/kg [97,98]. Fortunately, certified products rarely exceed gluten contents above 100 mg/kg but may contribute to an increased inadvertent intake of gluten.

Patients with CD following a GFD should be aware of numerous composite foods and medicines that contain hidden sources of vital gluten, which is frequently used as an additive to improve product quality. Composite foods, which increase the risk of gluten contamination, are ubiquitous, for example, soups and sauces at restaurants, coffee creamers at cafeterias, ice cream at ice cream parlors, and sausages at butcher shops. Eating at workplaces, schools, hospitals, assistive living facilities, and while visiting other people is an additional risk factor for hidden gluten intake. Inadvertent gluten intake via hidden channels can exceed the allowed amount by ten-fold or more. To prevent gluten intake, patients should study the label in case of prepacked products, where gluten has to be indicated as an allergen according to the Codex Standard 1–1985. In the case of unpacked products, patients should ask for information about gluten content.

Surprisingly, little is known about the quantity of gluten that is accidentally ingested by patients despite a GFD. Previous results of GIP analyses in stools and urine were used to estimate the amount of gluten consumed by patients with CD following a GFD for at least one year [99]. A total of 74 adults and adolescents (≥13 years old) were invited for a follow-up visit, in which stool and blood samples were collected. The computed daily mean gluten consumption for adults and adolescents measured in stool was 244 mg, for older children (4–12 years old) it was 387 mg, and for younger children (0–3 years old) it was 155 mg. The computed daily mean gluten consumption measured in urine was 363 mg for adults and adolescents and 316 mg for children. Individual data showed that a small portion of patients consumed more than 600 mg gluten on a daily basis. The analysis of GIP in stools from 64 pediatric patients with CD was used to estimate gluten intake at diagnosis and after 6, 12, and 24 months on a GFD [49]. The mean gluten exposure dropped from 5543 mg/day at diagnosis to 144 mg/day at 6 months on a GFD and then increased to 606 mg/day by 24 months. Recently, Silvester et al. [68,69] confirmed, in a period of 10 days, that 67% of patients with CD showed gluten exposure, and that the excretion kinetics were highly variable among individuals. The results demonstrated that most patients with CD can, in actuality, only attain a gluten-reduced diet, and gluten exposure is common, intermittent, and usually silent.

In addition, one of the promising advances to improve adherence to diet in patients with CD are the LFT Nima ™ (Nima Labs, Inc, San Francisco, CA, USA) and EZ Glutent ™ gluten sensors (ELISA Technologies, Inc, Gainesville, FL, USA), devices that have been developed to integrate food processing, gluten detection, interpretation results, and data transmission into a portable consumer device.

In conclusion, cross-contamination of gluten-free products with gluten is likely to be the main cause of inadvertent non-adherence to a GFD. The amount of gluten in certified gluten-free-labeled foods is normally low (rarely above 100 mg/kg). In contrast, naturally gluten-free foods as well as composite foods and medicines with hidden gluten may contain gluten amounts far above 100 mg/kg and are, therefore, serious risk factors for patients’ health. Stools and urine analyses have shown that patients with CD on a GFD are at risk for gluten consumption exceeding the allowed amounts by ten-fold or more. Non-adherence, caused by contaminated naturally gluten-free and composite foods, is rarely identified by dieticians and questionnaires; therefore, a promising advance could be the determination of gluten by consumers.

#### 2.3.2. Knowledge of GFD

Knowledge of a GFD and gluten-free foods is one of the most significant facilitators of GFD adherence identified in the literature. For example, knowledge that a strict GFD avoids immediate reactions and prevents long-term complications is a primary reason for dietary adherence. However, many individuals with CD exhibit significant deficits in their knowledge of a GFD and the gluten contents of foods, as exemplified by the following studies. A total of 5912 members of the two Canadian Celiac Associations filled in a questionnaire regarding knowledge of a GFD among other items [100]. When asked to review a list of 15 foods and ingredients and to identify those that were not allowed on a GFD, only 49% correctly identified all seven non-allowed items, and only an additional 33% correctly identified six of the seven non-allowed items. In another study, 82 adult Canadian patients with CD, having a medium of 6 years GFD experience, were asked to find gluten-containing foods among 17 different common foods [95]. None of the participants identified the potential gluten content of all the foods, and only 25 participants identified at least 14 foods correctly. A total of 104 adult Italian patients with CD completed a questionnaire comprising 31 statements on CD in general and foods appropriate in a GFD [65]. The patients’ knowledge was generally poor (only one patient answered all the questions correctly) and was significantly associated with poor GFD adherence.

Reliable, up-to-date, and comprehensive education could play an important role in improving knowledge and GFD adherence. Dietary and psychological counselling, for example through health professionals and dieticians, can essentially increase compliance with a GFD [20]. Studies of patients with CD, recruited from a CD clinic in New Delhi, showed that repeated counselling of patients with CD following a GFD remarkably increased the level of adherence [101]. At the beginning of the study, only 53% of subjects maintained excellent or good adherence to a GFD. After 6 months of repeated counselling, the level of adherence increased to 92%. The evaluation of 1832 US adults with CD (19–65 years old) revealed a highly significant association between dietary adherence to a GFD (indicated by a higher CDAT score) and having visited a healthcare provider [102].

Visiting healthcare providers may cause improved adherence, because they provide patients with a better understanding of how to implement the diet and appreciation of the diet’s importance. However, striking deficiencies in the quality of information and in the level of support that patients receive from their healthcare providers have been reported [33]. A total of 154 adult patients with CD judged the rate of adequate information and support provided by their healthcare providers as follows: dieticians, 63%; gastroenterologists, 57%; primary care physicians, 36%; and pharmacists, 23%. A large cross-sectional Canadian study, including 5912 adult patients with CD, resulted in low ratings of the usefulness of information provided by dieticians (52%), gastroenterologists (43%), and family physicians (25%) [100]. As confidence in treatment advice impacts GFD adherence, education of healthcare professionals should be improved substantially.

Apart from communication with healthcare providers, patient advocacy groups, and other persons with CD, the Internet, print media, and cookbooks are the most commonly used sources of information about a GFD [95]. Several studies have demonstrated that membership of celiac societies is associated with a greater GFD adherence; they particularly offer practical advice and support regarding gluten-free foods and a GFD. Members are often a self-selected group of patients who may exhibit greater motivation to adhere to a GFD and have significantly better knowledge of gluten-free foods than non-members. Moreover, psychological support counselling seems to be able to increase GFD compliance (Addolorato et al. [103]. A cohort of 66 Italian patients with CD with state anxiety and current depression were randomized in two groups. In group A, psychological support was started at the beginning of a GFD, while group B was free of psychological support. A follow-up after six months revealed that the subjects of group A had a significantly higher rate of GFD compliance (39.4%) compared to group B (9.1%).

Different online programs have been shown to be effective in significantly improving adherence and represent a promising resource for individuals with CD who are struggling to achieve adequate GFD adherence. To test the effectiveness of an interactive online intervention, 189 Australian adults with CD were recruited and divided into a group receiving the intervention (*n* = 101) and a control group without intervention (*n* = 88) [104]. The primary outcome measure was GFD adherence during a three-month follow-up. The online intervention showed significantly improved GFD adherence in the intervention group relative to the control group. The effectiveness of a smartphone app (MyHealthyGut, Vancouver, BC, Canada) in helping adults self-manage CD was assessed by Dowd et al. [105]. The participants of the study reported high levels of app usability and were satisfied with the features of the app. The vast majority of participants reported improvements in GFD adherence, gastrointestinal symptoms, quality of life, self-regulatory efficacy, anxiety, and depression.

As home cooking is among the top challenges for patients with CD and a frequent cause of non-adherence, the feasibility and acceptability of a cooking-based education intervention was assessed [106]. A total of 12 adult US patients with CD participated in two intervention sessions (2 consecutive days, 4.5 h each), co-led by the center dietician and a trained chef, and completed a follow-up interview. At the 1-month follow-up, participants had significantly improved GFD adherence and quality of life scores. All the participants agreed that the intervention was helpful, promoted eating foods they otherwise would not have tried, and made them more informed about gluten sources when eating out.

In summary, lack of knowledge about a GFD and gluten-free foods is an important cause of inadvertent non-adherence. Future interventions to improve adherence to a GFD should include methods with very high specificity and sensitivity to monitor gluten exposure in patients with CD, enhanced dietary and psychological counselling by healthcare providers with expertise in CD, as well as the promotion of education by online training programs.

#### 2.3.3. Difficulties with Certified Gluten-Free Foods

Apart from the dilemmas experienced when eating out, travelling, and socializing with friends, limited availability, high costs, poor quality, and not-understanding the labeling of dietetic gluten-free foods are frequent reasons for breaking a strict GFD intentionally [107]. Previously, dietetic gluten-free foods were niche market products, available almost exclusively in health food shops, pharmacies, and through mail order companies. Over the past few decades, the market for certified gluten-free foods has grown enormously, and nowadays they are also offered in many supermarkets and online providers. Nevertheless, dietetic gluten-free foods are not available everywhere, and patients with CD still have difficulty finding gluten-free foods when shopping. Limited access to gluten-free meals in canteens, schools, and kindergartens or whilst travelling is an additional barrier to GFD adherence.

A survey on the availability of 10 wheat-based everyday foods and 10 corresponding gluten-free counterparts at 30 different stores in London revealed an average availability of 82% of gluten-free foods [108]. Regular supermarkets had a greater availability (90%), whereas budget supermarkets (9%) and corner shops (9%) had almost no gluten-free versions. The inspection across four venues and five US geographic regions demonstrated that the availability of certified gluten-free products varied by region and venue but remained limited compared to their wheat-based counterparts [109]. Availability was greatest (66%) in health food and upscale venues. Among 38 South Asian patients with CD living in the UK, 85% reported no gluten-free foods in their local Asian stores [92]. Regarding eating out and travelling, dining establishments are frequently unable to provide safe meals; thus, patients make mistakes on their GFD [110].

The higher costs of dietetic gluten-free foods are a further barrier to adherence. For example, the cost of gluten-free foods (*n* = 63) and their gluten-containing counterparts (*n* = 126) were compared in 12 different Austrian supermarkets [111]. On average, gluten-free products were substantially higher in cost, ranging from +205% (breakfast cereals) to +267% (bread and bakery products) compared to similar gluten-containing products. Original data on retail prices in four major UK supermarkets within the UK “Bread and Cereal” category showed that the average price of gluten-free products was increased by a factor of 1.9 compared to corresponding gluten-containing products [112]. In Italy, the gluten-free version of pasta, the traditional staple food, was sold in supermarkets with an average price equal to more than twice that of conventional pasta [113]. A premium price was particularly found for the following attributes: small packages, brands that specialized in gluten-free products, content in fiber, and the presence of quinoa as an ingredient. A Greek study compared the cost of gluten-free products from supermarkets and pharmacies with the cost of their conventional counterparts [114]. All the supermarket gluten-free products, except for one, were more expensive by 22 to 334%, and all the pharmacy gluten-free products were more expensive by 88 to 476%. The weekly economic burden of a GFD, calculated for one person, ranged from EUR 12 to 28 per week. Gluten-free products, purchased across five geographic US regions and four venues in 2018, were 183% more expensive than their wheat-based counterparts [109]. In comparison to a study in 2006, the cost of gluten-free products has declined from 240 to 183%.

Adherence to a GFD is often associated with receiving gluten-free foods on prescription. In an English study, 375 adult patients with CD who were, in part, supported by the prescription of gluten-free foods, completed a CDAT to measure their dietary adherence [61]. Of the patients not receiving gluten-free foods on prescription, 62% were classified as non-adhering to a GFD compared with 42% of those receiving gluten-free foods on prescription. Paul et al. [115] suggested that in resource-limited settings, medical professionals should be creative in formulating cheaper and locally sourced gluten-free options in close co-operation with the dieticians, thereby ensuring the availability of gluten-free food items at affordable prices and the improvement of GFD adherence.

Patients are frequently unsatisfied with the quality of dietetic gluten-free foods, for example, due to poor flavor, taste, texture, and mouthfeel. In particular, the replacement of wheat bread and other baked goods, pasta, and beer by gluten-free substitutes is one of the most critical aspects of a GFD. Despite the improved quality of gluten-free breads in the last number of years, most products on the market are still described as low-quality products: gluten-free breads often have a low volume, pale crust, crumbly texture, bland flavor, and high rate of stalling, and gluten-free pasta products have an inferior texture [116]. The taste and flavor of gluten-free beer, made with alternative ingredients such as sorghum, millet, or buckwheat are not acceptable to many patients with CD. Novel strategies for the production of high-quality gluten-free beers, made from ultra-low gluten barley lines [117] or enzymatically detoxified barley malts [118], are currently in development and may contribute to improved compliance with a GFD.

Not understanding food labels is frequently associated with poorer dietary adherence. To investigate whether patients with CD can identify gluten-free foods based on product labeling, 25 different food items were presented to 144 adult US patients at 6, 12, and 24 months after CD diagnosis [119]. The median overall accuracy scores were 84, 96, and 84% at 6, 12, and 24 months, respectively. An examination of 375 adult patients with CD from the UK revealed that 73% of those who reported not understanding food labels were classified as non-adhering to a GFD compared with 45% who understood food labels [61]. A combined cross-sectional survey, including 972 ethnically different patients with CD residing in the UK, confirmed that there were substantial issues with the understanding of food labels that impacted adherence to a GFD [92].

In summary, numerous reasons are responsible for intentionally breaking a GFD. In particular, the limited availability, high costs, and poor quality of certified gluten-free products mislead patients to consume corresponding gluten-containing products. The non-ability to read and interpret food labels is another cause of concern, as it leads to inadvertent GFD non-adherence.

#### 2.3.4. Individual Factors

A number of studies have reported a broad spectrum of individual factors that impact adherence to a GFD in patients with CD, including gender, age, ethnicity, education, and mental health conditions [20,26]. The influence of gender on adherence rate has been judged differently. A systematic review, presented in 2013, reported no difference between men and women [89], whereas a large study of 2018 demonstrated that males are more adherent than females [62]. In contrast, a multicenter clinical trial studied the adherence of children and adults with CD to a GFD (*n* = 188) by measuring stool GIP [45]. When further stratified by gender, GFD adherence was found to be closely related to the patient’s gender in certain age groups. More males ≥13-years old had positive GIP stools compared with females in the same age group (60% vs. 31.5%, respectively, *p* = 0.034). The higher proportion of non-adherent male patients compared with females could be attributed to milder symptoms found in men or to stricter self-control over the diet in women. Regarding the age of the patients, the majority (85.7%) of celiac children between zero and three years of age had stools negative for GIP. Among those ≥13 years old, the proportion rose up to 39.2% with positive GIP. Altogether, these data show how increasing control over the diet could yield an increase in dietary adherence, as demonstrated by the four-fold greater adherence seen in children ≤3 years old. They have strong parental control over their diet, but no social pressure as compared with the adherence of adolescent males who are under little parental control but are subject to strong social influences. An Indian study of 134 children with CD found that the percentage of compliant children dropped from 76% in children aged 2–5 years to 41% in children above 9 years of age [44]. These results were in accordance with a previous Swedish study, which reported adherence rates of 93% at 12 years of age, decreasing to 76% in the 15–17 years age group [120]. In comparison, patients diagnosed later in life had relatively good adherence. The examination of GFD adherence among 35 biopsy-proven Finnish patients with CD aged over 50 years revealed that 27 patients (77%) maintained a strict diet, 5 patients (14%) had occasional transgressions less often than once a month, and 3 patients (9%) did not start a GFD [121]. In a cohort of Italian patients with CD aged over 65 years (*n* = 59), adherence to a GFD was 90% [122].

For adolescents with CD, adherence to a GFD is linked to many difficulties, and non-adherence is common even among those aware of the risks. The majority of transgressions occur intentionally at home or at parties. The reasons for non-adherence are manifold. Adolescents are aware of being different from others when maintaining a GFD, which often requires discussions and special requests [123]. Public eating can produce stigmatizing experiences in adolescence, and thus, dietary non-adherence can be understood in terms of dealing with GFD concealment. The absence of symptoms after consuming a small amount of gluten, the absence of peer acceptance, and even more often troublesome diet administration are further common reasons for non-adherence [45,47].

The “Prague consensus” focused on the GFD difficulties during the transition of CD-affected adolescents to adulthood, which presents a fragile and high-risk period for intentional and inadvertent gluten intake [124]. Although young children with CD may adhere to a GFD because of parental influence, the situation remains complex in adolescents. Several mechanisms for improving GFD adherence among youth have been identified, including regular CD engagement with an experienced multidisciplinary team, electronic tool utilization, and awareness of accurate resources for self-guided education [125].

Asian patients with CD living in Western countries may find it more difficult to adhere strictly to a GFD for a number of reasons [91]. If their command of the local language is poor, their understanding of food labeling will be compromised. They often live within an extended family setting, which puts increased pressure on them to comply with their cultural norms and, therefore, neglect a GFD. In addition, making Asian foods with naturally gluten-free materials can be very difficult.

Individuals with a high level of education have been shown to have higher GFD adherence. A study on long-term GFD adherence among 355 adult US patients with CD demonstrated that the level of education differed significantly between the subjects with adequate and inadequate adherence [57]. A higher level of education was associated with adequate adherence (*p* = 0.002), even after controlling for household income. Furthermore, a significant inverse correlation was found between the CDAT score and education level.

The circumstances in which the temptation to break the diet is most likely are practical in nature: being busy, having limited time or a break from usual routine, and difficulty in finding gluten-free foods when eating away from home [126]. Physical and emotional factors may be being physically unwell, tired, lacking energy, and bored as well as being stressed, upset or down, and emotionally exhausted. The overall health of patients and their adherence levels were shown to be highly correlated [94]. The health-related quality of life score obtained by patients with CD who reported perfect GFD adherence was found to be significantly higher than that obtained by patients with CD who reported unintended lapses and patients with CD who reported intentional lapses. A systematic review with meta-analysis, including eight cross-sectional studies, demonstrated a moderate association between poor GFD adherence and self-reported depressive symptoms, but further studies are needed to confirm this association [127].

The relationship between the strength of motivation and GFD adherence has been shown in several studies. For example, a Treatment Self-Regulation Questionnaire and a CDAT, administered to 433 South Italian people with CD, aged between 18 and 79 years, demonstrated that motivation strongly correlated with GFD adherence [128]. Poor adherence can be associated with low self-regulation, self-efficacy, facilitation, support, and psychological distress, social fear, depression, or frequent self-control lapses. Therefore, it is necessary to consider these factors in the treatment of individuals with CD. Studies of 200 North American adults with CD revealed that self-compassion predicted stricter adherence to a GFD both directly and indirectly through self-regulatory efficacy [129]. These findings indicate that self-compassion and concurrent self-regulatory efficacy are important cognitions in understanding adherence to a GFD.

In conclusion, individual facilitators and barriers concerning GFD adherence are manifold and include various socio-demographic factors such as education, age, and ethnicity as well as mental health conditions such as motivation, self-efficacy, and depression. It is necessary to consider these factors in the counselling of individuals with CD to improve GFD adherence. However, the number of studies that have investigated this aspect is still low, and future research is urgently needed.

## 3. Conclusions

The rates of GFD adherence among patients with CD reported in the literature are highly variable and are determined by the degree of adherence, the methods to assess it, and the barriers to its implementation. Inadvertent dietary lapses are distinctly more frequent than intentional lapses. For these reasons, adherence to a GFD by patients with CD have been reported to be far away from the optimal. The methods for evaluating the adherence to a GFD are comprised of a dietary questionnaire, serological test, or clinical symptoms; however, none of these methods generate either a direct or an accurate measure of dietary adherence. A small-bowel biopsy is the “gold-standard” method for CD diagnosis. However, according to most clinical guidelines, its role in the follow-up of patients is limited to cases involving a lack of clinical response or the recurrence of symptoms. A promising advancement is the development of tests that measure GIP in stools and urine. The cross-contamination of gluten-free products with gluten is one of the main causes of inadvertent non-adherence. Therefore, adequate nutritional counselling as well as an assessment technique for a GFD are necessary for patients diagnosed with CD in order to help in ascertaining dietary compliance and to target the most suitable intervention during follow-up and prevent the risk of possible complications long term.

## Figures and Tables

**Table 1:** Studies on the rate of adherence to GFD in children, adolescents, and adults in different countries. Anti-TGA, anti-tissue transglutaminase antibody; CD, celiac disease; CDAT, Celiac Dietary Adherence Test; GFD, gluten-free diet; GIP, gluten immunogenic peptides; NCGS, non-celiac gluten sensitivity.

	Country	Characteristics of Patients	*n*	Method	% Adherence to a GFD	References
**Children**	India	CD, >6 months on GFD	134	Questionnaires	66	Garg and Gupta, 2014 [44]
	Spain	CD	114	GIP	79	Comino et al., 2016 [45]
	Spain	CD and healthy subjects	65	GIP	55	Moreno et al., 2017 [46]
	Poland	CD, >2 years on GFD	102	Questionnaires Serology (anti-TGA)	67	Czaja and Bulsa, 2018 [47]
	Slovak Republic	CD	325	Questionnaires	69	Rimárová et al., 2018 [48]
	Australia	CD	151	BIAGI	86	Wessels et al., 2018 [43]
	Australia	CD	151	Standardized dietary interview	48	Wessels et al., 2018 [43]
	Spain	CD, >2 years on GFD	64	GIP	75	Comino et al., 2019 [49]
	Spain	CD	80	GIP	92	Fernández-Miaja et al., 2020 [50]
	Spain	CD, >6 months on GFD	43	GIP	65	Roca et al., 2020 [51]
	Italy	CD	200	BIAGI and Serology (anti-TGA)	84–100	Sbravati et al., 2020 [52]
**Teenagers**	Brazil	CD, >1 years on GFD	35	Questionnaires Serology	80	Rodrigues et al., 2018 [53]
	Italy	CD, >2 years on GFD	58	Questionnaires	36	Zingone et al., 2018 [54]
	Sweden	CD, >5 years on GFD	70	CDAT	86	Johansson et al., 2019 [55]
**Teenagers and adults**	Spain	CD	74	GIP	61	Comino et al., 2016 [45]
**Adults**	Italy	CD, >1 years on GFD	65	CDAT	82	Galli et al., 2014 [56]
	United States, US	CD, 10 years on GFD	355	CDAT	76	Villafuerte-Galvez et al., 2015 [57]
	Mexico	CD and NCGS, >3 years on GFD	80	CDAT	58	Ramírez-Cervantes et al., 2016 [58]
	Canada	CD, >4 years on GFD	222	CDAT	56	Silvester et al., 2016a [59]
	Canada	CD, 6 months on GFD	105	CDAT	91	Silvester et al., 2016b [60]
	Spain	CD and healthy subjects	69	GIP	52	Moreno et al., 2017 [46]
	United Kingdom, UK	CD, >3 years on GFD	375	CDAT	53–81	Muhammad et al., 2017 [61]
	Australia and New Zealand	CD, >6 months on GFD	5310	Online surveys	61	Halmos et al., 2018 [62]
	Italy	CD, >5 years on GFD	750	Questionnaires Symptoms	90–91	Tovoli et al., 2018 [63]
				Serology		
	Argentina	CD, >2 years on GFD	44	GIP	75	Costa et al., 2019 [64]
	Italy	CD, >1 years on GFD	104	CDAT	65	Paganizza et al., 2019 [65]
	Israel	CD, >4 years on GFD	301	BIAGI	82	Dana et al., 2020 [66]
	Spain	CD, 7 years on GFD	271	CDAT	72	Fueyo-Diaz et al., 2020 [67]
	Canada	CD	18	GIP	23	Silvester et al., 2020a; 2020b [68,69]
	Spain	CD, >2 years on GFD	77	GIP	42	Ruiz-Carnicer et al., 2020 [24]
	Argentina	CD, >2 years on GFD	53	GIP	11–62	Stefanolo et al., 2020 [70]
	Spain	CD	76	GIP	21	Fernández-Bañares et al., 2021 [23]

## Data Availability

Not Applicable.
